# Phenotypic Resemblance to Neuropsychiatric Disorder and Altered mRNA Profiles in Cortex and Hippocampus Underlying *IL15R*α Knockout

**DOI:** 10.3389/fnins.2020.582279

**Published:** 2021-02-03

**Authors:** Yi He, Yuxin Yu, Yanan Li, Weicheng Duan, Zuoli Sun, Jian Yang, Abba J. Kastin, Weihong Pan, Yan Zhang, Kang Wang

**Affiliations:** ^1^The National Clinical Research Center for Mental Disorders, Beijing Key Laboratory of Mental Disorders, Beijing Anding Hospital, Capital Medical University, Beijing, China; ^2^Advanced Innovation Center for Human Brain Protection, Capital Medical University, Beijing, China; ^3^Department of Endocrinology, Union Hospital, Tongji Medical College, Huazhong University of Science and Technology, Wuhan, China; ^4^Department of Gastrointestinal Surgery, Seventh Affiliated Hospital, Sun Yat-sen University, Shenzhen, China; ^5^Department of Forensic Medicine, Tongji Medical College, Huazhong University of Science and Technology, Wuhan, China; ^6^Pennington Biomedical Research Center, Baton Rouge, LA, United States; ^7^BioPotentials Consult, Sedona, AZ, United States; ^8^Department of Clinical Laboratory, Union Hospital, Tongji Medical College, Huazhong University of Science and Technology, Wuhan, China

**Keywords:** *IL15R*α, RNA-seq, gene set enrichment analysis, neuroscience, overlap analysis, lipid metabolism

## Abstract

**Background:**

Previous studies of the functions of *IL15R*α have been limited to immune activities and skeletal muscle development. Immunological factors have been identified as one of the multiple causes of psychosis, and neurological symptoms have been described in *IL15R*α knockout (KO) mice. Seeking to explore possible mechanisms for this in the *IL15R*α^–/–^ mouse brain, we analyzed gene expression patterns in the cortex and hippocampus using the RNA-seq technique.

**Methods:**

*IL15R*α KO mice were generated and littermate wildtype (WT) mice were used as a control group. A Y-maze was used to assess behavior differences between the two groups. The cortex and hippocampus of 3-month-old male mice were prepared and RNA-seq and transcriptome analysis were performed by gene set enrichment analysis (GSEA).

**Results:**

Compared with the WT group, *IL15R*α KO animals showed higher speed in the novel arm and more entrance frequency in the old arm in the Y-maze experiment. GSEA indicated that 18 pathways were downregulated and 13 pathways upregulated in both cortex and hippocampus from the GO, KEGG, and Hallmark gene sets. The downregulated pathways formed three clusters: respiratory chain and electron transport, regulation of steroid process, and skeletal muscle development.

**Conclusion:**

*IL15R*α KO mice exhibit altered expression of multiple pathways, which could affect many functions of the brain. Lipid biosynthesis and metabolism in the central nervous system (CNS) should be investigated to provide insights into the effect of *IL15R*α on psychosis in this murine model.

## Introduction

Psychosis is a group of complex diseases with various unknown etiologies. There is a large economic burden to patients with disease such as schizophrenia and depression, associated with increased direct healthcare costs, lost productivity, changes in employment status, and suicide (Jin and Mosweu, 2017; Amos et al., 2018). Resulting sociological issues include violence, crime, and other problems. A focus of psychiatric research has been the relationships between the central nervous, immune, and endocrine systems (Steinberg et al., 2015). Depression and schizophrenia patients exhibit dysregulation in their immune function (Arolt et al., 2002), and IL-1β, IL-2r, IL-6, and TNF-α have been identified as biomarkers of schizophrenia (Upthegrove et al., 2014).

The immunomodulatory cytokine interleukin-15 (IL-15) exhibits functional similarities with IL-2 and is ubiquitously expressed in the normal CNS as well as other organs (He et al., 2010a). Compared with IL-2/IL-2Rα, IL-15/IL-15Rα is a more broadly expressed bio-regulator that affects a wider range of target cell populations, giving it the ability to regulate the homeostasis and growth of various non-immune cell types and tissues (Budagian et al., 2006). *IL-2R*α KO mice have manifestations of autoimmunity and 25% die of severe anemia (Willerford et al., 1995). By contrast, *IL-15R*α KO mice exhibit several unique alterations in immune cell development and function without increased mortality (Lodolce et al., 1998).

mRNA for *IL-15R*α isoforms is widely expressed in microglia, astrocytes, and neurons, and it has a recognized major signaling role in cerebral function (Lee et al., 1996; Hanisch et al., 1997; Kurowska et al., 2002). *IL-15R*α KO mice show hyperactivity, depressive-like behavior, reduced anxiety, and impaired memory (He et al., 2010a; Pan et al., 2013). Recent studies reported a rare loss-of-function *IL-15R*α mutation in a schizophrenic patient and his siblings by sequencing—the clinical manifestations were similar to *IL-15R*α KO mice (Pan et al., 2019). KO mice have further abnormalities such as a leaner body composition with lower fat (He et al., 2010b), altered skeletal motor activation, elevation of body temperature, and increased energy expenditure (He et al., 2010b).

How lipid metabolism can influence CNSs is attracting a rising interest. Altered lipid metabolism was linked to many neurological disorders, such as autism spectrum disorder and schizophrenia (Khan et al., 2002; Bell et al., 2004; Chauhan et al., 2004). The *IL-15* was proved to inhibit lipid deposition in murine 3 T3-L1 preadipocytes, and global *IL-15R*α KO mice were observed with lower body fat accompanied by a series of neurological disorders (Budagian et al., 2006; He et al., 2010a; Loro et al., 2015). In this study, we sought a holistic view of the potential molecular mechanisms or pathways within the brain affected by the deletion of *IL-15R*α. With the help of NGS technology, we studied the transcriptome in the cortex and hippocampus of *IL15R*α^–/–^ mice. GSEA was used to explore common mechanisms of the two brain regions, with validation by qRT-PCR. In addition, we describe connections among enriched pathways and cluster them into three themes.

## Materials and Methods

### Animal Maintenance

Male *IL15R*α^–/–^ mice were purchased from the Jackson Laboratory (003723) and were crossed to C57BL/6 females from Beijing Charles River Laboratories. After six generations of reproduction, heterozygous mice were identified and maintained for the next experiments. All mice were housed under 12/12 h light/dark conditions with free access to food and water in the animal facility of Capital Medical University. Littermate homozygous mice were obtained by pairing heterozygous mice. Genotyping was performed to differentiate *IL15R*α^–/–^ mice from WT animals. All experiments were conducted under 3R principles (reducing, reusing, and recycling) according to local ethical and safety rules. Animal work was approved by the Animal Use and Care Committee at Capital Medical University.

### Animal Behavior Observations in a Y-Maze

The Y-maze (470 × 160 × 460 mm) consisted of three arms (start arm, novel arm, and the other arm) at an angle of 120° to each other, interconnected by a central zone. Mice underwent two trials: (1) with the novel arm blocked, mice were introduced to the start arm and allowed to freely explore the start arm and other arm for 10 min and (2) after a 1-h interval, mice were placed into the start arm again and had access to the start arm, novel arm, and other arm for 5 min. All trials were conducted in the same environment without human interference. The Y-maze was cleaned with a 5% alcohol solution between each trial to eliminate possible biasing effects from odor cues left behind. All trials were recorded by the camera above the maze, and subsequent video recordings were analyzed by the Supermaze animal behavior analysis system. Two-tailed unpaired Student’s *t*-tests were applied using IBM SPSS Statistics for statistical data analysis, with *p*-value < 0.05 considered significant.

### Total RNA Extraction and Library Construction

The cortex and hippocampus were microdissected from 3-month-old *IL15R*α^–/–^ and WT brains (*n* = 3) and then soaked and stored in TRIzol Reagent (Invitrogen, Catalog 15596026). RNA extraction was conducted according to the manufacturer’s instructions and quantification was performed using Qubit (Invitrogen). Library construction and sequencing were conducted by Igenecode Technology, Beijing, China. Following the manufacturer’s instructions, mRNA was captured and isolated by magnetic beads coupled with Oligo (dT). The fragmented mRNA was then converted into double-stranded (ds) cDNA, ligated with a sequencing adapter, and amplified by PCR.

### RNA Sequencing

After RNA quality control, RNA-seq was carried out with the HiSeq sequencing strategy using the Illumina second-generation high-throughput sequencing platform. Low-quality data were removed, and subsequently the filtered reads were aligned to the *Mus musculus* genome (GRCm38) using HISAT (Kim et al., 2015). The mapped reads were counted and converted to FPKM (Mortazavi et al., 2008) by RSEM (Li and Dewey, 2011).

### Correlation Analysis

Biological replicate samples were grouped into one module in which FPKMs of each gene were averaged. The processed data were imported into GraphPad Prism and subjected to linear regression and Pearson correlation analysis, comparing cortex and hippocampus in both mutant and control groups.

### Differential Gene Expression Analysis and qRT-PCR Validation

The matrix including raw counts from mutant and control groups was imported into R (v.3.6.3). The DESeq2 (Love et al., 2014, 2) (v.1.26.0) package was applied to analyze whether the expression pattern had changed due to *IL15R*α KO and to identify significantly up-/downregulated genes in cortex or hippocampus. Only genes with an absolute value of log2 (fold change) ≥ 1 and *p*-value < 0.05 were regarded as differentially expressed between mutant and control groups.

Quantitative real-time PCR (qRT-PCR) was performed to evaluate and validate the expression pattern of DEGs in the cortex and hippocampus (Supplementary Table S1). cDNA was synthesized using the cDNA Reverse Transcription Kit (TIANGEN). All reactions were run in duplicate PCR amplification cycles in a real-time PCR instrument ABI7500 (ABI). Target mRNA was quantified relative to the β-*actin* gene as a reference molecule and analyzed with two-tailed unpaired Student’s *t*-tests to assess statistical significance of gene expression levels between the *IL15R*α^–/–^ and control groups in cortex or hippocampus.

### Gene Set Enrichment Analysis

Pathway enrichment analysis was implemented in the GSEA software (v.4.0.3) by comparing expression patterns between the *IL15R*α^–/–^ and control groups. Annotated gene sets were downloaded from the MSigDB (Liberzon et al., 2011) and included H (Hallmark gene sets) (Liberzon et al., 2015), KEGG (KEGG subset of CP), BP (GO biological process), CC (GO cellular component), and MF (GO molecular function). Only pathways with nominal *p*-values < 0.05 in both cortex and hippocampus were taken into consideration. The subsets were visualized by bubble diagrams created in R (v.3.6.3). Overlapped matrix of subsets was uploaded into Circos (Krzywinski et al., 2009) (v.0.63-9^1^) to perform online visualization.

### Network Visualization and Analysis

To construct a union enrichment map, both cortex and hippocampus data were imported into Cytoscape (Doncheva et al., 2019) (v3.8.0) software and clusters were obtained after pruning the merged networks. ClusterMaker2 (Morris et al., 2011) (v.1.3.1) and AutoAnnotate (Kucera et al., 2016) (v.1.3.3) plugins helped to build clusters based on the topological structure of visualized networks and a summary of these annotations was generated by the WordCloud (Oesper et al., 2011) (v.3.1.3) plugin as a tag cloud over the clusters.

## Results

*IL15R*α expression data from brains of both schizophrenia patients and healthy controls were extracted from the SZDB database (Supplementary Table S2) (Wu et al., 2017). There is a significant association of hippocampal *IL15R*α expression with schizophrenia (*p* = 8.32 × 10^–5^), with a significant FDR (*p* = 0.01046). Schizophrenia patients seem to have higher *IL15R*α expression levels in the hippocampus than controls (Supplementary Figure S1).

**FIGURE 1:**
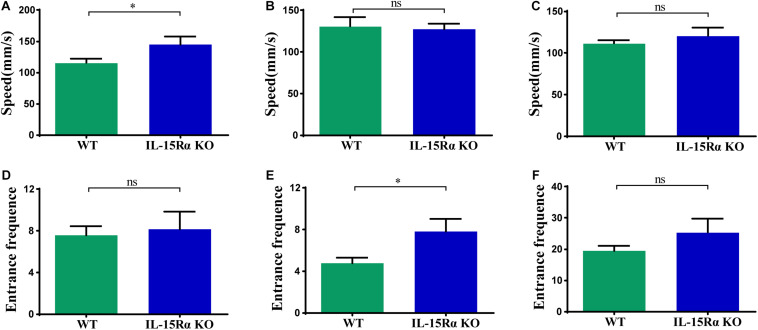
Speed and entrance frequency behavior abnormalities after *IL15R*α knockout. **(A)** Histogram presents the average speed of IL15Rα mice and wildtype mice in novel arm. **(B)** Histogram summarizes the average speed of IL15Rα mice and wildtype mice in old arm (start arm and the other arm). **(C)** Histogram reveals the average speed of IL15Rα mice and wildtype mice in total arm (novel arm, start arm, and the other arm). **(D)** Histogram presents the entrance frequency of IL15Rα mice and wildtype mice in novel arm. **(E)** Histogram summarizes the entrance frequency of IL15Rα mice and wildtype mice in old arm (start arm and the other arm). **(F)** Histogram reveals the entrance frequency of IL15Rα mice and wildtype mice in total arm (novel arm, start arm, and the other arm). Average speed is computed from total distance traveled in certain region divided by total time in certain region. The results are depicted as the mean ± SD from eight biological replicates in IL15Rα group and 12 biological replicates in control group. The level of significance is calculated by two-tailed unpaired Student’s *t*-test. *p < 0.05.

The Y-maze test was chosen for behavioral testing to assess cognitive functions of mice after *IL15R*α KO. Utilizing the innate tendency to explore a novel environment (Deacon and Rawlins, 2006), we recorded the distance, time, speed, and entrance frequency in each arm, respectively. *IL15R*α^–/–^ mice showed a higher speed in the novel arm (Figure 1A) and more entrance frequency in the old arm (Figure 1E). With regard to the total arm (novel arm, start arm, and other arm), the two groups showed no significant difference in speed or entrance frequency (Figures 1C,F).

**FIGURE 2:**
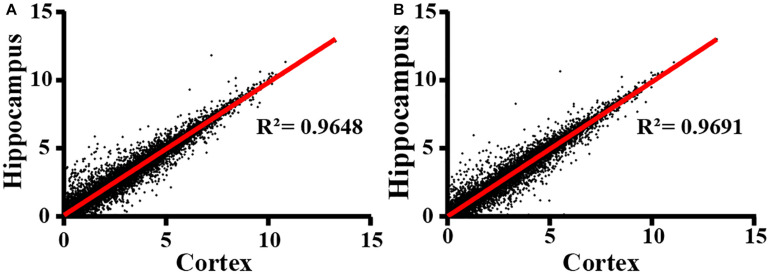
Correlation of RNA expression profiles between cortex and hippocampus. Cortex and hippocampus are computed for sample correlation by Pearson’s correlation analysis in *IL15R*α^–/–^ group **(A)** and control group **(B)**, respectively. Pearson’s correlation coefficient is represented by *R*^2^ values.

Next, we extracted total RNA samples from cortex and hippocampus in adult WT control and *IL15R*α KO mice. Transcriptional profiles were assessed by NGS RNA-seq. After data quality control, we compared expression patterns between cortex and hippocampus in both the *IL15R*α^–/–^ group (Figure 2A) and control group (Figure 2B), which showed Pearson’s correlations of 0.9648 and 0.9691, respectively. This finding reveals similar expression patterns between cortex and hippocampus. Therefore, we compared enrichment outcomes of cortex and hippocampus and selected overlapping features for further analysis in the following steps, to provide explanations of transformation in these two brain regions.

### DEG Identification and qPCR Validation

**FIGURE 3:**
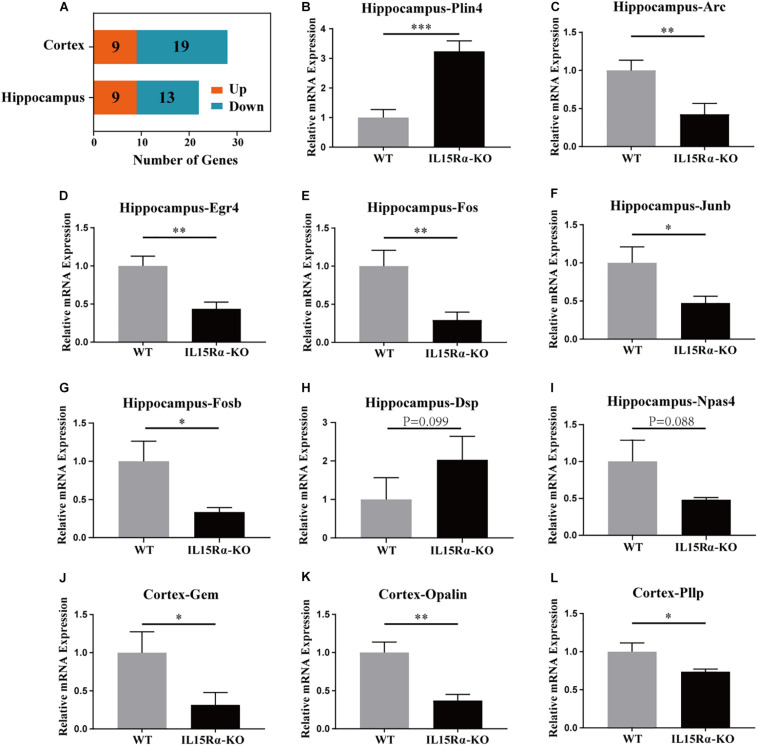
DEG analysis in cortex and hippocampus and qRT-PCR validation of certain DEGs. **(A)** Stacked diagram reveals the amount of up-/downregulated DEGs in cortex and hippocampus. The genes with absolute value of log2 (fold change) ≥ 1 and *p*-value < 0.05 between *IL15R*α^–/–^ group and control group are defined as DEGs. DEGs possessing log2(fold change) > 0 are considered to be significantly upregulated, whereas the other are significantly downregulated. **(B–I)** Six DEGs (*Plin4*, *Arc*, *Egr4*, *Fos*, *Fosb*, and *Junb*) in hippocampus are evaluated to be differently expressed between *IL15R*α^–/–^ group and control group by qRT-PCR. **(J–L)** Three DEGs (*Gem*, *Opalin*, and *Pllp*) in cortex are validated to be differently expressed between *IL15R*α^–/–^ group and control group by qRT-PCR. Expression is determined by the relative quantification method with β-*actin* gene as internal reference. Relative mRNA expression is calculated from three biological replicates and depicted as the mean ± SD after normalizing to average expression in control group. The level of significance is calculated by two-tailed unpaired Student’s *t*-test. *p < 0.05; **p < 0.01; ***p < 0.001.

Compared with control mice, 28 genes were expressed differentially in the *IL15R*α^–/–^ group in the cortex and 22 in the hippocampus, with FDR < 0.05 for both—these included nine upregulated and 19 downregulated genes or nine upregulated and 13 downregulated genes, respectively (Figure 3A). DEGs were listed (Supplementary Table S3), and 11 interesting listed genes were chosen to validate the transcript sequencing results, including three genes in the cortex and eight in the hippocampus. Expression changes were detected by qPCR using reverse transcript cDNA from total RNA samples (Figures 3B–L). These 11 genes are involved in lipid metabolism, oligodendrocytic function, GTP binding, and cytoskeletal function. The results demonstrate that transcription levels of *Gem*, *Opalin*, and *Pllp* are downregulated in the cortex and *Arc*, *Egr4*, *Fos*, *Fosb*, *Dsp*, *Npas4*, and *Junb* are downregulated and *Plin4* upregulated in the hippocampus. Quantitative PCR analysis was in accordance with the NGS results (Supplementary Tables S3, S4).

### GSEA Generated Gene Set Enrichment and Functional Annotation

**FIGURE 4:**
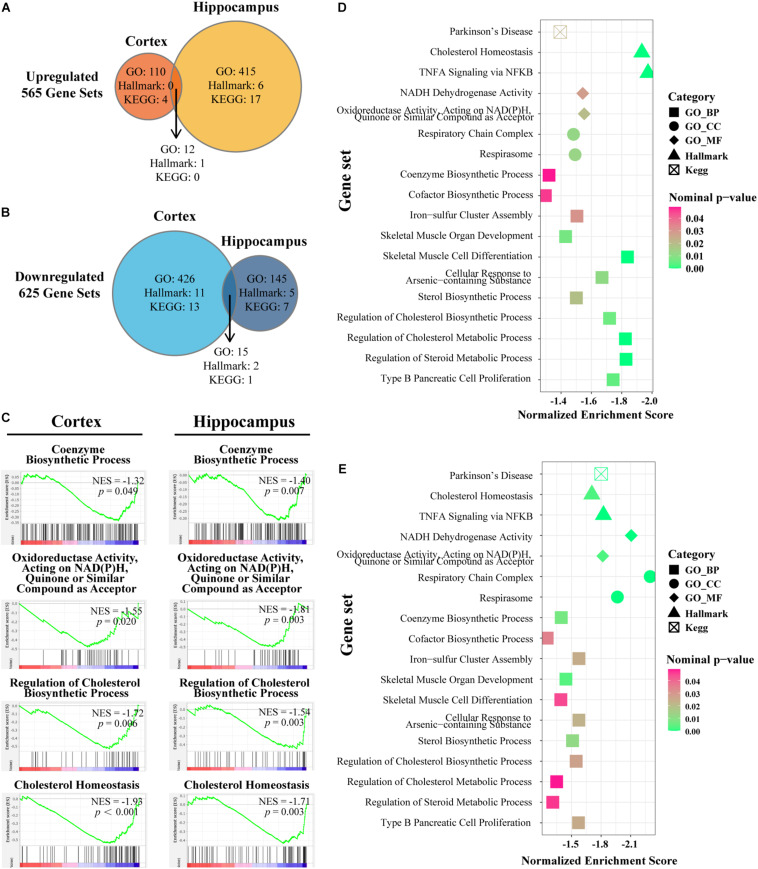
Gene set enrichment analyses in cortex and hippocampus and the intersection of the enriched pathways. **(A,B)** Venn diagram depicts the amount of significantly up-/downregulated pathways with nominal *p*-value < 0.05 in the cortex or hippocampus of *IL15R*α^–/–^ samples. Enriched pathways are retrieved from following sets of genes: H (Hallmark gene sets), KEGG (KEGG subset of CP), BP (GO biological process), CC (GO cellular component), and MF (GO molecular function). Overlapped areas in the diagram count the commonly contributing pathways in both cortex and hippocampus. **(C)** Enrichment plot provides a graphical view of the enrichment score for pathways chosen from downregulated overlapped pathways. **(D)** Bubble diagram conveys the core characters of certain pathways in cortex. **(E)** Bubble diagram presents the state of certain pathways in hippocampus. The shape, color, and abscissa value of points indicate the category of gene sets, magnitude of nominal *p*-value, and normalized enrichment score, respectively. Pathways are selected from overlapped downregulation.

As a computational method that determines whether a defined set of genes shows statistically significant concordant differences between two biological states, GSEA focuses on groups of genes that share common biological function or regulation (Subramanian et al., 2005). We chose three widespread and frequently used gene sets from MSigDB, namely, Hallmark, GO, and KEGG gene sets for further enrichment analyses by GSEA. The procedure was performed in cortex and hippocampus separately and the overlap region identified with the criteria *p*-value < 0.05. In total, 625 pathways were identified as downregulated and 565 as upregulated in the three pre-defined gene sets (Figures 4A,B). Based on previous studies, IL-15 shares many biological properties with interleukin-2 (IL-2) (Grabstein et al., 1994). Furthermore, IL15Rα and IL2Rα are tightly linked in both mouse and humans with respect to gene size and organization (Anderson et al., 1995; Tagaya et al., 1996). In accordance with an expectation of a compensation mechanism, significantly overlapping upregulated enriched pathways were mapped to the regulation, biosynthesis, or production of *IL-2* and inflammatory response or negative regulation of immune system processes (Supplementary Table S5). Next, we confirmed four existing representative enriched pathways based on the aforementioned results: coenzyme biosynthetic process, oxidoreductase activity acting on NADPH quinone or similar compound as acceptor, regulation of cholesterol biosynthetic process, and cholesterol homeostasis. Both cortex and hippocampus showed significant enrichment at the head of the targeted gene sets with NES < −1 (Figure 4C). In the next section, we concentrated our attention on overlapping downregulated enriched pathways and cluster terms of both cortex and hippocampus were identified (Figures 4D,E). A total of 18 downregulated pathways were composed of 11 GO-BP ontology, two GO-CC ontology, two GO-MF ontology pathways, two Hallmark pathways, and one KEGG pathway (Supplementary Table S5). For the *IL15R*α^–/–^ group, cortex showed highly NES terms on cholesterol homeostasis (Hallmarks), skeletal muscle cell differentiation (GO-BP), and regulation of steroid or cholesterol biosynthetic or metabolic process (GO-BP) (Figure 4D). At the same time, pathways related to catalysis of an oxidation–reduction reaction in NADH or NADPH (GO-MF), respiratory chain activity (GO-CC), and cholesterol homeostasis (Hallmarks) were markedly enriched in the hippocampus (Figure 4E).

### Overlapped Pathways Connection Analysis and Unified Construction of Enrichment Map

**FIGURE 5:**
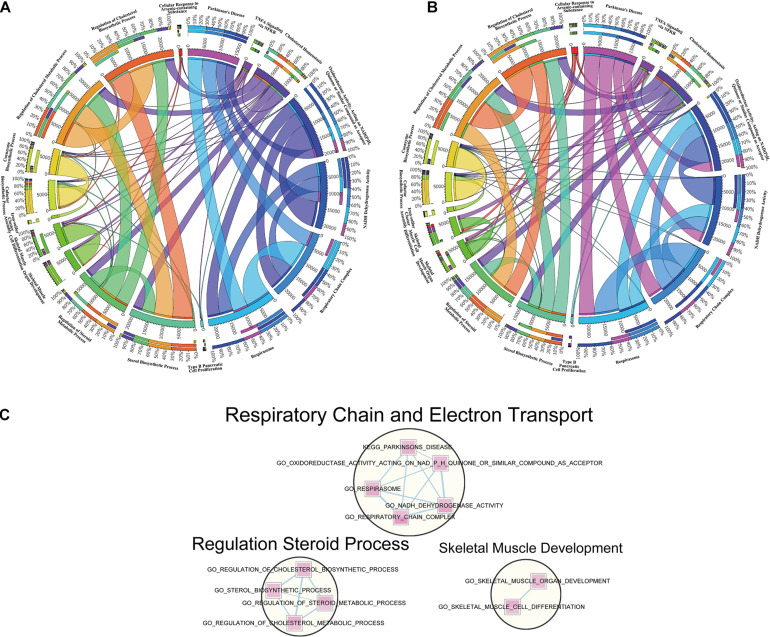
Leading-edge analysis of downregulated overlapped pathways. **(A)** Circos diagram presents a circular visualization of connection patterns in cortex within the 18 pathways chosen from overlapped downregulation. **(B)** Circos diagram utilizes a circular layout to facilitate the display of interaction patterns in hippocampus within the 18 pathways chosen from overlapped downregulation. The width of line represents various degrees to the relevance among pathways. **(C)** Enrichment networks reveal the important clusters achieved from the 18 overlapped downregulated gene sets. The pathways in circles represent the functionally associated sub-modules in the overall networks based on the topological structures, with the functional annotation displayed above.

To make a specific cross-examination between the 18 overlapped pathways in cortex and hippocampus, we defined a new gene set composed of these gene set names and specific continents from MSigDB of the BROAD Institute (Liberzon et al., 2011, 0). To extract the core members of the chosen pathways and explore connections between them, leading-edge analysis was used to grasp leading-edge subsets. The link intensity among the 18 pathways was drawn based on the similarity of contributors. Cortex (Figure 5A) and hippocampus (Figure 5B) showed analogous connection patterns within the 18 pathways but differed when analyzed in detail. There were complicated connections of different strengths (represented by width of the lines) in the circle, showing close or alienated relationships among pathways. Both cortex and hippocampus samples revealed dense connections from TNF-α signaling via NFκB pathways and many others. For the respiration-related and electron transport pathways, such as respirasome, respiratory chain complex, or NADPH dehydrogenase activity, hippocampus samples had more connective links with cofactor biosynthetic process and coenzyme biosynthetic process. Another difference is a larger connection weight between sterol biosynthetic with regulation of cholesterol biosynthetic and metabolic process, in cortex samples.

Designed for visualization networks among enriched pathways, “enrichment map” is a cytoscape plugin that has erected a bridge between GSEA software and cytoscape. An enrichment map helps to identify interesting pathways and themes (Reimand et al., 2019). Here, we detected clusters of enriched pathways based on the visualized enrichment map of the 18 pathways (Figure 5C). The largest cluster, labeled “respiratory chain and electron transport,” was composed of five pathways, followed by the second biggest cluster labeled “regulation steroid process” and the last cluster labeled “skeletal muscle development.”

## Discussion

With an extensive number of genes reported to be associated with various diseases, the genetic mechanisms of neurological abnormalities are regarded as highly heterogeneous. Accumulated evidence confirms that *IL15R*α insufficiency results in several disorders, including but not limited to immunodeficiency, skeletal muscle variation, and a series of neurological symptoms (Lodolce et al., 1998; He et al., 2010a,b; Pan et al., 2013; O’Connell et al., 2015). In this study, we first performed Y-maze tests to examine mouse behavioral properties after *IL15R*α KO. Y-maze is a behavioral test based on the willingness of rodents to explore new environments. The task-involved brain regions include the hippocampus, septum, basal forebrain, and prefrontal cortex. Overall, *IL15R*α^–/–^ mice showed a little higher speed and a little more entrance frequency (without significance) throughout the testing process (Figures 1C,F). However, *IL15R*α^–/–^ mice moved faster in the novel arm (Figure 1A) and moved equally in the old arm (Figure 1B). Although previous studies have demonstrated that lack of *IL15R*α influences muscle mitochondrial structure and function and results in improvement of athletic performance (Loro et al., 2018), *IL15R*α^–/–^ mice only exhibited higher speed in novel environments without the old arm. We suppose this was due to the high neuroexcitability that brings greater alertness in the new environment, rather than changes in skeletal muscle. In addition, compared with the similar entrance frequency of the novel arm (Figure 1D), *IL15R*α^–/–^ mice return to the old arm more frequently (Figure 1E). Mice are born with curiosity to explore new environments and the Y-maze test is designed to detect it. In a former anxiety evaluation trial, *IL15R*α KO mice showed reduced anxiety both in the open field test and elevated plus maze test (Wu et al., 2010). Among the identified DEGs, the immediate–early gene *Arc* was reported as dynamically regulated in the hippocampus (Guzowski et al., 2006). An increase of *Arc* has been observed in the fear memory (Clifton et al., 2017). In this study, the expression of Arc was found to decrease in *IL15R*α KO mice, supporting the behavior with less anxiety. The increasing curiosity may result in a higher speed in the novel arm, and the lowered anxiety may bring mice more frequently returning to the old arm, similar to the increase in the inner grids crossed in the open field test. Our previous study discovered that the *IL15R*α KO mice did worse in memory retention than the controls 1 week after the initial trial by stone 14-unit T-maze (He et al., 2010a). However, mouse performance in a Y-maze indicated no deficit in short-term memory after 1 h of the initial trial. The difference between the two experiments may be due to the time interval after training, the different rationale of the two experiments, or to genetic differences. Next, we aimed to explore mechanisms of neurological abnormality in *IL15R*α insufficiency and identify new gene sets and pathways in *IL15R*α^–/–^ mouse brain. Three-month-old male littermate mice housed in a barrier environment were selected to exclude interference from genetic background, age, and feeding conditions. This can ensure a more reliable analysis of differences caused by *IL15R*α deletion compared with WT gene transcription. Our analysis clearly demonstrates that the cortex and hippocampus response to *IL15R*α deletion involves alterations in multiple pathways related to the respiratory chain, along with electron transport, regulation of steroid processes, skeletal muscle development, and regulation and biosynthesis or production of *IL-2*.

High-throughput sequencing technology can produce big data but has the disadvantage of inaccuracy. To verify the transcriptome results, qRT-PCR was used to quantitate the expression levels of 11 DEGs (Supplementary Tables S3, S4). Among our validated DEGs, *Fos* is a family of transcription factors consisting of four members: *FOS*, *FOSB*, *FOSL1*, and *FOSL2*. The *Fos* family is involved in biological processes including neurogenesis, neuron differentiation, and neuron development. *Npas4* is also a kind of transcriptional regulator, which is involved in a wide range of physiologic and developmental events (Ooe et al., 2004). *Egr1*, *Egr2*, and *Egr4* belong to the Egr family of transcriptional regulatory factors, which is implicated in neuronal plasticity. These identified abnormalities in transcription factors indicated an association with numerous mental health disorders in *IL15R*α KO mice, including less anxiety or the memory disorders. Subsequently, GSEA was utilized to assist analysis and interpretation of the long lists of gene expression data into more easily interpretable biological pathways (Powers et al., 2018). Both cortex and hippocampus have been widely reported to be involved in psychosis, including autism and schizophrenia (Schultz and Andreasen, 1999; Carper and Courchesne, 2005; Duan et al., 2020). Exploring the transcriptomic changes in these organs in the *IL15R*α insufficiency mouse model will help to achieve a better understanding of the molecular pathways involved in this disease and benefit future novel diagnosis and treatment methods.

Our GSEA screening characterized the alterations of expression profiles and identified overlapping pathways enriched in both cortex and hippocampus (Figure 4). We found that skeletal muscle organ development and cell differentiation in the two brain regions were enriched, consistent with previous studies (Loro et al., 2018). Another enrichment module was related to steroid processes, including regulation of steroid biosynthesis and metabolism, and cholesterol biosynthetic and metabolic processes. Sterol lipids are an essential class of lipids in all mammals, synthesized in many forms with the major form being cholesterol, and are of particular importance to the brain (Tracey et al., 2018). Abnormal lipid metabolism has been identified as hallmarks of neurodevelopmental and neurodegenerative disorders (Hebbar et al., 2017), for example, dysfunction in lipid metabolism and clearance were observed to be implicated in Alzheimer’s disease pathogenesis (Di Paolo and Kim, 2011; Visan, 2017). Considering the fact that neurons have a lower capacity for cholesterol synthesis (Zhang and Liu, 2015), sterol or cholesterol disorders may induce abnormalities in the brain. Cholesterol synthesis or metabolism requires acetyl CoA and electron input at multiple steps, utilizing both NADH and NADPH as the electron source (Porter, 2015). Indeed, this was exactly the pathway we enriched (Figures 4C–E) accompanied by steroid processes pathways. Furthermore, the pathway cluster of chain and electron transport involved in respiratory was also enriched (Figure 5C).

There were also some findings to be revisited. Steroid and cholesterol biosynthetic and metabolic pathway was more enriched in cortex than hippocampus in *IL15R*α^–/–^ mice (Figure 5A), whereas the pathway related to respiratory chain or electron transfer showed more prominence in the hippocampus (Figure 5B). More downregulated pathways were enriched in the cortex; by contrast, more upregulated pathways were enriched in the hippocampus (Figures 4A,B). These subtle differences may indicate divergence of mechanisms in the two brain regions, which is worth further investigation in future research.

To summarize, we analyzed gene expression patterns in *IL15R*α KO mice by RNA-seq and validated some of the DEGs by qPCR. Transcriptional changes in overlapping pathways emerged and clustered into three groups. The data from *IL15R*α^–/–^ mice led us to propose that sterol biosynthetic and metabolic in the brain may be a key factor in schizophrenia-like etiology.

## Data Availability Statement

The datasets presented in this study can be found in online repositories. The names of the repository/repositories and accession number(s) can be found below: https://db.cngb.org/cnsa/, CNP0001191.

## Ethics Statement

The animal study was reviewed and approved by the Ethics Committee of Animal Use and Care Committee at Capital Medical University.

## Author Contributions

YH, ZS, and JY designed and conducted the experiments. YH provided biological samples. YL and YZ supported animal care. YH and KW conducted the statistical analysis. YY and YL analyzed and visualized high-throughput sequencing data. YY, WD, and KW cooperated in writing the original draft. AK and WP helped to revise the article and proposed constructive opinions. All authors approved the original draft of the article.

## Conflict of Interest

WP was employed by the company BioPotentials Consult. The remaining authors declare that the research was conducted in the absence of any commercial or financial relationships that could be construed as a potential conflict of interest. The reviewer XW declared a past co-authorship with several of the authors YH, ZS, JY, AK, and WP to the handling editor.

## Footnotes

1 http://circos.ca/

## Abbreviations

CNS: central nervous system
DEGs: differentially expressed genes
FDR: false discovery rate
FPKM: fragments per kilobase of exon model per million reads mapped
GO: gene ontology
GO-BP: gene ontology biological process
GO-CC: gene ontology cellular component
GO-MF: gene ontology molecular function
GSEA: gene set enrichment analysis
HISAT: hierarchical indexing for spliced alignment of transcripts
KEGG: Kyoto Encyclopedia of Genes and Genomes
KO: knockout
MSigDB: Molecular Signatures Database
NES: normalized enrichment score
NGS: next-generation sequencing
qRT-PCR: quantitative real-time polymerase chain reaction
RNA-Seq: whole transcriptome sequencing
RSEM: RNA-Seq by expectation-maximization
SZDB: schizophrenia database
WT: wildtype.
