# Supplementary material for: Phenotypic Resemblance to Neuropsychiatric Disorder and Altered mRNA Profiles in Cortex and Hippocampus Underlying IL15Rα Knockout
Source: Front Neurosci. 2021 Feb 3;14:582279. doi: 10.3389/fnins.2020.582279 (PMC7887313; doi:10.3389/fnins.2020.582279)
Supplement: Supplementary file 1 [file Table_1.DOCX]

**Supplementary of Mice neuropsychiatric disorders like phenotype and mRNA profiles in cortex and hippocampus underlying IL15Rα knockout**

**Figure legend**

**Supplementary Figure S1**. *IL15RA* expression profiles in the human hippocampus and prefrontal cortex

Box plot displays normalized *IL15RA* expression level in the hippocampus and prefrontal cortex between schizophrenia patients and controls. The box plot is extracted from SZDB 1.0 database.

| **Supplementary Table S1** Primer sequence designed for qRT-PCR in cortex and hippocampus of mice | |
| --- | --- |
| Gene | Primer Sequence (5'-3') |
| *Plin4* | Forward: AGCCCATGACAACTGAGG |
|  | Reverse: TCACCCAGACGGATGTAG |
| *Arc* | Forward: ATGACTATACCGTTAGCC |
|  | Reverse: CAAGACTGATATTGCTGA |
| *Egr4* | Forward: GGAGGCGACTTCTTGAGC |
|  | Reverse: CTGTAGCTGAGGCCCGAA |
| *Fos* | Forward: AGCCGACTCCTTCTCCAG |
|  | Reverse: TGGTGGAGATGGCTGTCA |
| *Junb* | Forward: AGGAACCGCAGACCGTAC |
|  | Reverse: CCACTTTGATGCGCTCCT |
| *Gem* | Forward: TGACAGCATGGACAGCGA |
|  | Reverse: TTCCCACATGTCCAGGAG |
| *Opalin* | Forward: CGAATACGACATCCTCTC |
|  | Reverse: GGTATCTCAGTCTCCTCTA |
| *Pllp* | Forward: CCACGGTTCTCTACATTACT |
|  | Reverse: GCAATCATCACCAGACAAG |
| *β-actin* | Forward: CACCACACCTTCTACAATGAG |
|  | Reverse: TACGACCAGAGGCATACAG |
| *Fosb* | Forward: CAGGAACCGTCGGAGGGA |
|  | Reverse: GGCGATCTCCGACTCCAG |
| *Dsp* | Forward: GAAGTCAGCAATCTACCAGTT |
|  | Reverse: CTCCTCCTCGCAGTCATT |
| *Npas4* | Forward: GAGTGTGAGCGAGCATCT |
|  | Reverse: ATCAGCATCCAGAGCAGAG |

| **Supplementary Table S2** IL15RA expression level in the human brain between case and control groups^a^ | | | | | | | | |  |
| --- | --- | --- | --- | --- | --- | --- | --- | --- | --- |
| Gene | Probe | Hippocampus | | |  | Prefrontal Cortex | | | |
|  |  | FoldChange | *P*-value | FDR |  | FoldChange | *P*-value | FDR |  |
| *IL15RA* | 207375_s_at | 1.22 | 8.32e-05 | 0.01046 |  | 1.05 | 3.31e-01 | 0.7392 |  |
| ^a^DatafromSZDB1.0 | | | | | | | | |  |

| **Supplementary Table S3** DEGs^a^ in cortex and hippocampus | | | | | | |  |
| --- | --- | --- | --- | --- | --- | --- | --- |
| Gene Name | Description | Cortex | |  | Hippocampus | | |
|  |  | log_2_(FoldChange)^b^ | adjusted P value |  | log_2_(FoldChange) | adjusted P value | |
| *Fosb* | FBJ osteosarcoma oncogene B | -1.26 | 8.77E-04 |  | -1.19 | 7.01E-09 | |
| *Xdh* | xanthine dehydrogenase | 1.25 | 8.92E-03 |  | 1.47 | 8.81E-04 | |
| *Plin4* | perilipin 4 | 1.36 | 3.03E-03 |  | 2.32 | 2.29E-06 | |
| *Hspa1a* | heat shock protein 1A | -1.32 | 4.85E-07 |  | -1.05 | 2.09E-07 | |
| *Aebp1* | AE binding protein 1 | -1.06 | 4.70E-02 |  |  |  | |
| *Fkbp5* | FK506 binding protein 5 | 1.00 | 1.28E-03 |  |  |  | |
| *Fosl2* | fos-like antigen 2 | -1.30 | 9.28E-05 |  |  |  | |
| *Gem* | GTP binding protein (gene overexpressed in skeletal muscle) | -1.99 | 6.90E-04 |  |  |  | |
| *Hspa1b* | heat shock protein 1B | -1.16 | 2.54E-03 |  |  |  | |
| *Inhba* | inhibin beta-A | 1.36 | 1.79E-02 |  |  |  | |
| *Klf2* | Kruppel-like factor 2 (lung) | -1.28 | 5.25E-04 |  |  |  | |
| *Klf4* | Kruppel-like factor 4 (gut) | -1.41 | 3.19E-02 |  |  |  | |
| *Sik1* | salt inducible kinase 1 | -1.17 | 1.53E-04 |  |  |  | |
| *Gadd45b* | growth arrest and DNA-damage-inducible 45 beta | -1.02 | 2.02E-02 |  |  |  | |
| *Myoc* | myocilin | -1.98 | 7.33E-04 |  |  |  | |
| *Nes* | nestin | -1.32 | 1.28E-04 |  |  |  | |
| *Dusp1* | dual specificity phosphatase 1 | -1.03 | 6.99E-03 |  |  |  | |
| *Hif3a* | hypoxia inducible factor 3, alpha subunit | 1.21 | 4.70E-02 |  |  |  | |
| *P3h4* | prolyl 3-hydroxylase family member 4 (non-enzymatic) | -1.17 | 8.92E-03 |  |  |  | |
| *Ppp1r14a* | protein phosphatase 1, regulatory (inhibitor) subunit 14A | -1.42 | 1.06E-02 |  |  |  | |
| *4930447C04Rik* | RIKEN cDNA 4930447C04 gene | -1.13 | 1.06E-02 |  |  |  | |
| *Car12* | carbonic anhydrase 12 | 1.46 | 2.01E-03 |  |  |  | |
| *Mcam* | melanoma cell adhesion molecule | -1.07 | 1.21E-02 |  |  |  | |
| *Plekhg4* | pleckstrin homology domain containing, family G (with RhoGef domain) member 4 | 1.46 | 3.56E-03 |  |  |  | |
| *Utp14b* | UTP14B small subunit processome component | -1.61 | 7.58E-04 |  |  |  | |
| *Rrp36* | ribosomal RNA processing 36 homolog (S. cerevisiae) | 1.73 | 5.94E-03 |  |  |  | |
| *Opalin* | oligodendrocytic myelin paranodal and inner loop protein | -1.80 | 1.25E-04 |  |  |  | |
| *Fam19a1* | family with sequence similarity 19, member A1 | 1.15 | 2.54E-03 |  |  |  | |
| *Arc* | activity regulated cytoskeletal-associated protein |  |  |  | -1.10 | 4.99E-02 | |
| *Egr1* | early growth response 1 |  |  |  | -1.24 | 2.36E-02 | |
| *Egr2* | early growth response 2 |  |  |  | -2.86 | 1.65E-09 | |
| *Egr4* | early growth response 4 |  |  |  | -1.51 | 8.66E-03 | |
| *Fos* | FBJ osteosarcoma oncogene |  |  |  | -1.88 | 6.14E-04 | |
| *Junb* | jun B proto-oncogene |  |  |  | -1.32 | 1.07E-03 | |
| *Spp1* | secreted phosphoprotein 1 |  |  |  | 1.07 | 4.08E-02 | |
| *Thbs4* | thrombospondin 4 |  |  |  | 1.16 | 1.71E-05 | |
| *Stk32b* | serine/threonine kinase 32B |  |  |  | -1.22 | 3.62E-02 | |
| *Gpr88* | G-protein coupled receptor 88 |  |  |  | 1.44 | 1.77E-02 | |
| *Rps4l* | ribosomal protein S4-like |  |  |  | -1.26 | 3.32E-06 | |
| *Snurf* | SNRPN upstream reading frame |  |  |  | -1.04 | 6.38E-07 | |
| *Dsp* | desmoplakin |  |  |  | 1.13 | 1.14E-02 | |
| *Cacng5* | calcium channel, voltage-dependent, gamma subunit 5 |  |  |  | 1.03 | 1.22E-02 | |
| *Slc17a6* | solute carrier family 17 (sodium-dependent inorganic phosphate cotransporter), member 6 |  |  |  | 1.17 | 5.94E-04 | |
| *Npas4* | neuronal PAS domain protein 4 |  |  |  | -1.67 | 6.88E-08 | |
| *Cdh19* | cadherin 19, type 2 |  |  |  | 1.01 | 3.05E-02 | |
| *Orai3* | ORAI calcium release-activated calcium modulator 3 |  |  |  | -1.39 | 2.21E-02 | |
| ^a^ Genes with absolute value of log2(FoldChange) ≥1 and adjusted P value <0.05 between IL15Rα-/- group and control group were identified as significant DEGs.  ^b^ DEGs possessing log2(fold change) >0 are considered to be significantly up-regulated, whereas the other are significantly down-regulated. | | | | | | |  |

| **Supplementary Table S4** Expression patterns of the 11 mRNAs selected for RT-qPCR validation | | | | | | | |
| --- | --- | --- | --- | --- | --- | --- | --- |
| Region | Gene | RT-qPCR | |  | RNA-seq | |  |
|  |  | Fold Change^a^ | *P*-value |  | Fold Change | *P*-value |  |
| Cortex | *Gem* | 0.32 | 0.021 |  | 0.25 | 0.001 |  |
|  | *Opalin* | 0.37 | 0.002 |  | 0.29 | 0.000 |  |
|  | *Pllp* | 0.74 | 0.019 |  | 0.50 | 0.001 |  |
| Hippocampus | *Plin4* | 3.24 | 0.001 |  | 5.01 | 0.000 |  |
|  | *Arc* | 0.42 | 0.007 |  | 0.47 | 0.050 |  |
|  | *Egr4* | 0.44 | 0.003 |  | 0.35 | 0.009 |  |
|  | *Fos* | 0.29 | 0.006 |  | 0.27 | 0.001 |  |
|  | *Junb* | 0.47 | 0.016 |  | 0.40 | 0.001 |  |
|  | *Fosb* | 0.34 | 0.013 |  | 0.44 | 0.000 |  |
|  | *Dsp* | 2.03 | 0.099 |  | 2.18 | 0.011 |  |
|  | *Npas4* | 0.48 | 0.088 |  | 0.31 | 0.000 |  |
| ^a^Fold change is calculated as the ratio of the expression patterns under the treatment condition to the control condition, describing how much a quantity changes going from the control condition to the treatment condition. | | | | | | | |

| **Supplementary Table S5** Significantly up/down regulated gene sets^a^ in both cortex and hippocampus. | | | | | | | | |
| --- | --- | --- | --- | --- | --- | --- | --- | --- |
| Regulation | Category | Gene Sets Name | SIZE | Cortex | |  | Hippocampus | |
|  |  |  |  | NES^b^ | nominal P value |  | NES | nominal P value |
| Down Regulation | GO biological process | Type B pancreatic cell proliferation | 20 | -1.72 | 0.003 |  | -1.56 | 0.041 |
|  |  | Regulation of steroid metabolic process | 107 | -1.82 | 0.001 |  | -1.31 | 0.043 |
|  |  | Regulation of cholesterol metabolic process | 53 | -1.82 | 0.000 |  | -1.38 | 0.043 |
|  |  | Regulation of cholesterol biosynthetic process | 40 | -1.70 | 0.006 |  | -1.58 | 0.008 |
|  |  | Sterol biosynthetic process | 71 | -1.50 | 0.026 |  | -1.52 | 0.006 |
|  |  | Cellular response to arsenic-containing substance | 19 | -1.63 | 0.010 |  | -1.57 | 0.031 |
|  |  | Skeletal muscle cell differentiation | 61 | -1.84 | 0.000 |  | -1.40 | 0.025 |
|  |  | Skeletal muscle organ development | 152 | -1.43 | 0.014 |  | -1.42 | 0.014 |
|  |  | Iron-sulfur cluster assembly | 21 | -1.52 | 0.032 |  | -1.58 | 0.026 |
|  |  | Cofactor biosynthetic process | 218 | -1.31 | 0.035 |  | -1.27 | 0.014 |
|  |  | Coenzyme biosynthetic process | 140 | -1.33 | 0.039 |  | -1.38 | 0.022 |
|  | GO cellular component | Respirasome | 79 | -1.52 | 0.008 |  | -1.90 | 0.000 |
|  |  | Respiratory chain complex | 69 | -1.48 | 0.024 |  | -2.37 | 0.000 |
|  | GO molecular function | Oxidoreductase activity, acting on NAD(P)H, quinone or similar compound as acceptor | 45 | -1.57 | 0.009 |  | -1.78 | 0.000 |
|  |  | NADH dehydrogenase activity | 37 | -1.54 | 0.012 |  | -2.08 | 0.000 |
|  | Hallmark gene sets | TNFA signaling via NFKB | 188 | -1.99 | 0.000 |  | -1.81 | 0.000 |
|  |  | Cholesterol homeostasis | 71 | -1.92 | 0.000 |  | -1.72 | 0.003 |
|  | KEGG subset of CP | Parkinson's disease | 103 | -1.40 | 0.030 |  | -1.79 | 0.000 |
| Up Regulation | GO biological process | Interleukin-2 Biosynthetic Process | 21 | 1.54 | 0.025 |  | 1.73 | 0.007 |
|  |  | Interleukin-6 Production | 127 | 1.37 | 0.000 |  | 1.41 | 0.030 |
|  |  | Negative Regulation of Immune Effector Process | 99 | 1.36 | 0.047 |  | 1.63 | 0.000 |
|  |  | Negative Regulation of Immune Response | 126 | 1.39 | 0.005 |  | 1.53 | 0.003 |
|  |  | Negative Regulation of Immune System Process | 389 | 1.16 | 0.029 |  | 1.34 | 0.009 |
|  |  | Positive Regulation of Interleukin-2 Production | 30 | 1.55 | 0.032 |  | 1.65 | 0.005 |
|  |  | Regulation of Inflammatory Response | 292 | 1.26 | 0.000 |  | 1.45 | 0.004 |
|  |  | Respiratory Burst | 31 | 1.86 | 0.012 |  | 1.59 | 0.020 |
|  | GO cellular component | Endocytic Vesicle Lumen | 15 | 1.56 | 0.032 |  | 1.55 | 0.040 |
|  | GO molecular function | Immune Receptor Activity | 105 | 1.52 | 0.000 |  | 1.77 | 0.000 |
|  |  | Lipase Activity | 108 | 1.48 | 0.008 |  | 1.61 | 0.004 |
|  |  | Phospholipase Activity | 94 | 1.38 | 0.031 |  | 1.55 | 0.009 |
|  | Hallmark gene sets | Angiogenesis | 32 | 1.53 | 0.040 |  | 1.67 | 0.005 |
| ^a^ Gene sets withnominal P value <0.05 between IL15Rα-/- group and control group are determined as a significant variation.  ^b^NES refers to normalized enrichment score. NES >0 implies the up-regulation of gene sets, whereas NES <0 indicates the down-regulation of gene sets. | | | | | | | | |
