# Supplementary figures and images for: Phenotypic Resemblance to Neuropsychiatric Disorder and Altered mRNA Profiles in Cortex and Hippocampus Underlying IL15Rα Knockout
Source: Front Neurosci. 2021 Feb 3;14:582279. doi: 10.3389/fnins.2020.582279 (PMC7887313; doi:10.3389/fnins.2020.582279)

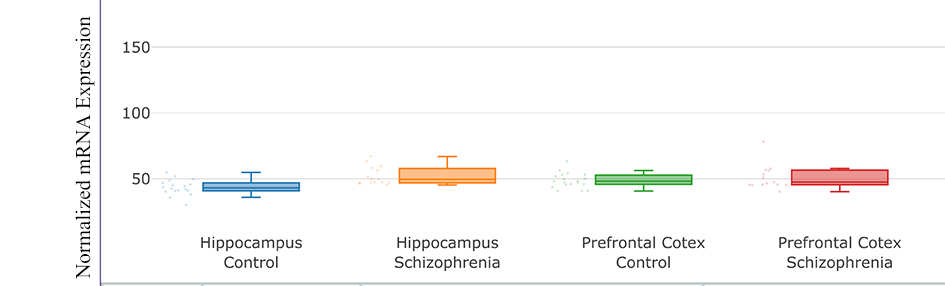

Supplement: Supplementary file 2 [file Image_1.TIF]
